# Somatosensory Abnormalities for Painful and Innocuous Stimuli at the Back and at a Site Distinct from the Region of Pain in Chronic Back Pain Patients

**DOI:** 10.1371/journal.pone.0058885

**Published:** 2013-03-15

**Authors:** Christian Puta, Birgit Schulz, Saskia Schoeler, Walter Magerl, Brunhild Gabriel, Holger H. W. Gabriel, Wolfgang H. R. Miltner, Thomas Weiss

**Affiliations:** 1 Department of Sports Medicine and Health Promotion, Friedrich Schiller University, Jena, Germany; 2 Center for Interdisciplinary Prevention of Diseases related to Professional Activities, Friedrich Schiller University, Jena, Germany; 3 Department of Biological and Clinical Psychology, Friedrich Schiller University, Jena, Germany; 4 Center for Biomedicine and Medical Technology Mannheim (CBTM), Ruprecht Karls University Heidelberg, Mannheim, Germany; University of Sao Paulo, Brazil

## Abstract

Chronic low back pain (CLBP) was shown to be associated with pathophysiological changes at several levels of the sensorimotor system. Changes in sensory thresholds have been reported but complete profiles of Quantitative Sensory Testing (QST) were only rarely obtained in CLBP patients. The aim of the present study was to investigate comprehensive QST profiles in CLBP at the painful site (back) and at a site distinct from their painful region (hand) and to compare these data with similar data in healthy controls. We found increased detection thresholds in CLBP patients compared to healthy controls for all innocuous stimuli at the back and extraterritorial to the painful region at the hand. Additionally, CLBP patients showed decreased pain thresholds at both sites. Importantly, there was no interaction between the investigated site and group, i.e. thresholds were changed both at the affected body site and for the site distinct from the painful region (hand). Our results demonstrate severe, widespread changes in somatosensory sensitivity in CLBP patients. These widespread changes point to alterations at higher levels of the neuraxis or/and to a vulnerability to nociceptive plasticity in CLBP patients.

## Introduction

Chronic low back pain (CLBP) is one of the major health problems, especially in industrialized countries. The costs of CLBP in the US alone are estimated to be at least $ 100 billions a year [1]. While there are many attempts to reduce costs by adequate therapy, treatment remains difficult because the pathophysiological changes in CLBP are still enigmatic.

CLBP was shown to be associated with several pathophysiological changes at various level of the sensorimotor system, including the cortical level (functional reorganization in somatosensory and motor regions [2]–[7]. CLBP patients were found to have a reduced mechanoreceptive and proprioceptive perception [8]–[11] as well as altered deep-tissue nociceptive perception [12]. In contrast to the detection levels, we recently found that CLBP patients exhibit a hypersensitivity to painful pin prick stimuli. Specifically, we found highly significant increases in pain rating especially to slight and moderate pin prick stimuli [13]. These changes were not only found at the painful area in the back but also at an unspecific extraterritorial region, i.e., at the hand demonstrating a generalized hypersensitivity to mechanical pin prick stimuli in CLBP patients. Obviously, there is a discrepancy between the increased thresholds for mechanical innocuous stimuli while the thresholds for mechanical noxious stimuli are decreased.

The hypothesis of generalized hypersensitivity to noxious stimuli seems to be important because of its possible impact for theoretical accounts to the pathophysiology of CLBP as well as to treatment options. To further evaluate this hypothesis, it seems necessary to investigate the whole profile of somatosensory sensations both at the painful site and at a site distinct from their region of pain. A well-established method for such an approach is the Quantitative sensory testing (QST) as introduced by the German research network for neuropathic pain (DFNS) [14], [15]. While the QST battery was primarily introduced to detect and to differentiate between different neuropathic syndromes, it also represents a tool for standardized somatosensory testing. Unexpectedly, there are only few studies using comprehensive QST in CLBP patients. Blumenstiel et al. [16] compared QST profiles of fibromyalgia patients with those of CLBP patients with a focus on fibromyalgia. Investigating QST of the hand and the back, they found significant changes on the back of CLBP patients with an increased threshold for vibration and a reduced threshold for pressure pain. While their data already demonstrate changes in QST profiles in CLBP patients that might be interpreted as generalized pain hypersensitivity, these authors did not specifically investigate QST at the painful site of CLBP patients but used sites similar to the examination in patients with fibromyalgia.

The aim of the present study was to investigate comprehensive sensory profiles of CLBP patients on the hand and on the painful site at the back and to compare these data with similar data in healthy controls (HC). So our primary hypothesis is that CLBP patients exhibit a generalized hypersensitivity to painful stimuli. We also hypothesize alterations for innocuous stimuli within the QST profile.

## Results

Results are presented with respect to the hypotheses, i.e. different to the usual representation of QST data by the DFNS. Here we analyzed data for pain thresholds (primary hypothesis) before presenting data for innocuous stimuli. Original data can be found in supplementary materials (Tables S1, S2, S3).

### Pain thresholds and related pain parameters within the QST profile

**Figure 1:**
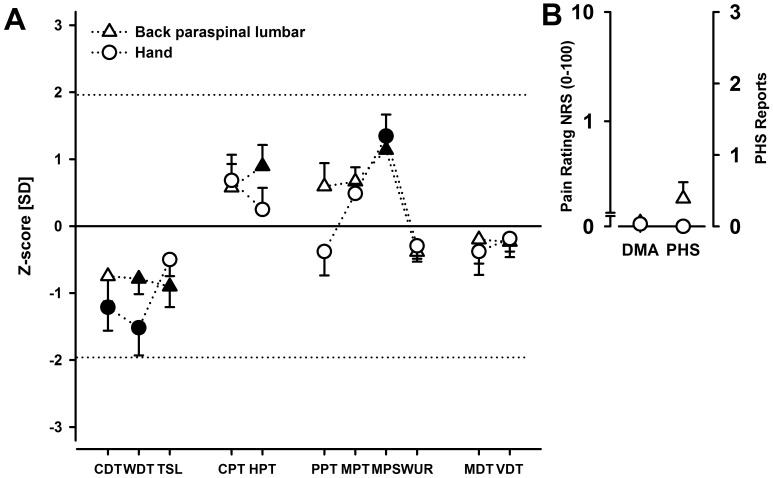
Quantitative sensory testing in chronic low back pain patients. Sensory profiles of QST parameters at the painful site (back paraspinal lumbar; triangles) and at a site distinct from the region of pain (hand; circles) are presented on the left side (**1 A**). Data for the QST parameters presented as z-scores (mean±SEM) with respect to healthy controls (HC). Filled symbols: significant difference for the patient group compared to HC (one-way ANOVA: P<0.05); open symbols: no significant difference. CDT, cold detection threshold; WDT, warm detection threshold; TSL, thermal sensory limen; CPT, cold pain threshold; HPT, heat pain threshold; PPT, pressure pain threshold; MPT, mechanical pain threshold; MPS, mechanical pain sensitivity; WUR, wind up ratio; MDT, mechanical detection threshold; VDT, vibration detection threshold. Paradoxical heat sensations (PHS) and dynamic mechanical allodynia (DMA) at the painful site (back paraspinal lumbar; triangles) and at a site distinct from the region of pain (hand; circles) are presented on the right side (**1 B**).

Analysing the pain thresholds and related pain parameters of the QST profile (see Figure 1 A), ANOVA with the factors Group (CLBP; HC), Region (hand; back), and Pain parameter (CPT; HPT; PPT; MPT; MPS; WUR) revealed a significant main effect of the factor Group (F(1,32) = 5.82; P<0.05). This main effect resulted from overall increased pain sensitivity for the CLBP group compared to HC (Figure 1 A). There was no main effect of factor Region (F(1,32) = 1.76; P>0.05). ANOVA also revealed a significant main effect of the factor Pain parameter (F(5,160) = 3.23; P<0.05; ε = 0.84). Post-hoc analysis revealed significantly lower values for the wind-up ratio WUR compared to the other pain thresholds (CPT; HPT; PPT; MPT; MPS; Duncan post-hoc test: all P<0.05; see Figure 1 A).

Beyond significant main effects, ANOVA with factors Group, Region, and Pain parameter revealed a significant interaction between Pain parameter and Group (F(5,160) = 3.23; P<0.05; ε = 0.84). Post-hoc contrasts revealed a significantly higher sensitivity for pinprick stimuli at threshold (MPT; P<0.05) and suprathreshold stimulus levels (MPS; P<0.01) for the CLBP patients compared to HC. Importantly, there were no significant interactions for Region x Group, Region x Pain parameter, or Group x Region x Pain parameter (all P>0.05). For detailed raw data - see Tables 1 and 2.

**Table 1:** Quantitative sensory testing (QST) in chronic low back pain patients (CLBP) and healthy controls (HC).

Back (paraspinal lumbar)		CLBP (N = 18) a	HC (N = 16) a	P-value CLBP vs. HC
CDT	Cold detection threshold (°C from BL; log) b	−1.94 (0.288±0.335)	−1.35 (0.130±0.210)	0.12
WDT	Warm detection threshold (°C from BL; log) b	2.33 (0.368±0.154)	1.75 (0.243±0.158)	<0.05
TSL	Thermal sensory limen (°C; log)	5.83 (0.766±0.209)	4.18 (0.621±0.161)	<0.05
CPT	Cold pain threshold (°C)	13.72±10.26	9.77±6.86	0.20
HPT	Heat pain threshold (°C)	41.83±3.43	44.12±2.55	<0.05
PPT	Pressure pain threshold (kPa; log)	152 (2.182±0.278)	197 (2.294±0.188)	0.19
MPT	Mechanical pain threshold (mN; log)	8.7 (0.939±0.189)	12.0 (1.078±0.208)	0.05
MPS	Mechanical pain sensitivity (pain rating 0–100; log)	3.19 (0.504±0.535)	0.83 (−0.083±0.515)	<0.01
WUR	Wind-up ratio (log)	2.48 (0.394±0.205)	3.30 (0.519±0.326)	0.20
MDT	Mechanical detection threshold (mN; log)	2.36 (0.373±0.468)	2.05 (0.312±0.305)	0.66
VDT	Vibration detection threshold (x/8)	5.02±0.87	5.23±0.89	0.50
DMA	Dynamic mechanical allodynia (pain rating 0–100; log)	0.113 (−0.947±0.146)	0.000 (−1.000±0.000)	0.15
	Number of subjects exhibiting DMA	3/18	0/16	0.27 ^c^
PHS	Paradoxical heat sensations (x/3)	0.39±0.98	0.00±0.00	0.12
	Number of subjects exhibiting PHS	3/18	0/16	0.27 ^c^
Hand (palmar)				
CDT	Cold detection threshold (°C from BL; log) b	−1.75 (0.243±0.174)	−1.26 (0.100±0.118)	<0.01
WDT	Warm detection threshold (°C from BL; log) b	1.87 (0.272±0.222)	1.20 (0.078±0.128)	<0.01
TSL	Thermal sensory limen (°C; log)	3.44 (0.536±0.164)	2.86 (0.457±0.158)	0.16
CPT	Cold pain threshold (°C)	6.70±6.41	4.00±3.95	0.16
HPT	Heat pain threshold (°C)	44.50±5.23	45.44±3.81	0.56
PPT	Pressure pain threshold (kPa; log)	238 (2.376±0.222)	209 (2.321±0.146)	0.41
MPT	Mechanical pain threshold (mN; log)	33.7 (1.527±0.249)	52.7 (1.722±0.399)	0.09
MPS	Mechanical pain sensitivity (pain rating 0–100; log)	1.86 (0.270±0.456)	0.66 (−0.178±0.334)	<0.01
WUR	Wind-up ratio (log)	2.14 (0.331±0.245)	2.62 (0.419±0.289)	0.35
MDT	Mechanical detection threshold (mN; log)	0.38 (−0.442±0.295)	0.30 (−0.517±0.199)	0.37
DMA	Dynamic mechanical allodynia (pain rating 0–100; log)	0.108 (−0.968±0.119)	0.101 (−0.995±0.02)	0.36
	Number of subjects exhibiting DMA	2/18	1/16	1.00 ^c^
PHS	Paradoxical heat sensations (x/3)	0.00±0.00	0.00±0.00	1.00
	Number of subjects exhibiting PHS	0/18	0/16	1.00 ^c^

a QST parameter, expressed as arithmetic mean±standard deviation (SD), or as geometric mean (log_10_mean±SD), or geometric mean retransformed from log_10_mean.

b Thermal detection thresholds are expressed as the difference from baseline temperature (BL = 32°C). ^c^ Yates corrected Chi-square.

**Table 2:** Quantitative sensory testing (QST) parameters assessed at the hand dorsum in chronic low back pain patients (CLBP) and healthy controls (HC).

Hand (dorsum)		CLBP (N = 18) a	HC (N = 16) a	P-value CLBP vs. HC
MPS	Mechanical pain sensitivity (pain rating 0–100; log)	2.22 (0.346±0.463)	0.81 (−0.092±0.429)	<0.01
VDT	Vibration detection threshold (x/8)	6.91±0.51	7.03±0.63	0.55
DMA	Dynamic mechanical allodynia (pain rating 0–100; log)	0.108 (−0.968±0.119)	0.000 (−1.000±0.000)	0.30
	Number of subjects exhibiting DMA	2/18	0/16	0.52 ^c^

a QST parameter, expressed as arithmetic mean±standard deviation (SD), or as geometric mean (log_10_mean±SD), or geometric mean retransformed from log_10_mean.

b Thermal detection thresholds are expressed as the difference from baseline temperature (BL = 32°C). ^c^ Yates corrected Chi-square.

Pain to light stroking tactile stimuli, i.e. dynamic mechanical allodynia (DMA), was very rare and only observed in CLBP patients (Table 1). It was found in 2 patients at each location of the hand and in 3 patients at the back (including the two patients with DMA at hand dorsum). Unexpectedly, innocuous cold stimuli at the back were found to elicit a painful sensation, i.e. paradoxical heat sensation (PHS; see Table 1), in 3 out of 18 patients (including one CLBP patient with DMA at the paraspinal lumbar back).

### Detection thresholds to innocuous stimuli within the QST profile

Mixed-model ANOVA with the factors Group (CLBP; HC), Region (hand, back), and Detection threshold (CDT, WDT, TSL, MDT, VDT) revealed a highly significant main effect of the factor Group (F(1,32) = 7.94; P<0.01). In contrast to analysis on pain parameters, CLBP patients showed significant lower sensitivity to innocuous stimuli (i.e. higher detection thresholds compared to HC - see Figure 1 A). No significant main effects were observed for factors Region (F(1,32) = 0.448; P>0.10) and Detection threshold (F(1,32) = 2.15; P>0.10; ε = 0.59). Furthermore all interactions were statistically not significant (all P>0.10). Table 1 demonstrates that the main effect of factor Detection threshold is mainly carried by the thermal detection thresholds.

### Comparison to reference data

We also compared our data with respect to the reference data from the German Research Network on Neuropathic Pain (DFNS). First, we compared the results of our HC group with the normative data. Results obtained at the palmar site of the hand in our HC group were in the range of the DFNS reference data (dorsum of the hand) as judged by the recommended quality self-control procedure with an average z-score of 0.11 for HC subjects was<95% confidence interval of DFNS reference data, recommended to be<0.25 [17], indicating good agreement with the DFNS standard. Second, we compared MPS at the hand dorsum (i.e., the reference site of the database) as the MPS represents one of the results of our study. MPS data of our HC group was almost perfectly normal as compared to the reference data (t = −0.001, P = 0.9). However, t*-*tests not only confirmed a significant group difference for MPS at the hand dorsum for the CLBP patients with respect to our HC group, but also with respect to the reference data (t = 2.95, P<0.01). The quality of data is underlined by the fact that z-score values for our CLBP patients normalized to our HC group, on the one hand, and normalized to the DFNS reference data [17], on the other hand, were significantly correlated (R^2^ = 0.976, P<0.001). Third, we compared the QST parameters between the affected area, i.e. the back, and the hand to distinguish between localized and generalized QST changes. When comparing these parameters for HC subjects, 11 out of 13 parameters were different at the back compared to the hand. This is in accordance with [16] also demonstrating differences between back and hand for control subjects in some QST parameters. So the differences indicate the necessity to provide separate normative data for some regions of the body, i.e. the back.

## Discussion

The aim of the present study was to investigate the profile of somatosensory changes occurring in CLBP patients at the affected painful region (back) and at a site distinct from the painful region (hand). CLBP patients exhibited an enhanced sensitivity to painful stimuli. Importantly, significantly enhanced pain sensitivity was also identified at the remote hand site. In addition, we also found a reduction of sensitivity to innocuous stimuli, especially for thermal detection thresholds. This phenomenon was also observed both at the hand and at the back.

### Pain thresholds and related pain parameters

Our data revealed significantly decreased pain thresholds in CLBP patients compared to HC subjects. Gain of function in the sense of decreased pain thresholds at the back has been report previously in CLPB patients for PPT [16] and for pinprick stimuli [13]. This result is in line with the expectation that CLPB patients have a lower pain threshold at the painful site even if our results include the pain thresholds as a main effect indicating that this is a more general phenomenon for the back. The difference in the profile compared to the Blumenstiel et al. study (2011) might lie in the exact site of examination and the control site. Blumenstiel et al. (2011) used cervicothoracal segments at the back as a control site in healthy controls and compared these results with examinations partially at the cervical, partially at the lumbar segments. Their study was primarily conducted to compare patients suffering from fibromyalgia with CLPB patients. Our study was conducted to investigate QST profiles in CLPB patients at the painful site (and at a site distinct from the painful region). Therefore, our finding at the back might well be more general with respect to the observed lower pain thresholds at the painful site.

Importantly, the pain thresholds observed at the hand in our patients showed very similar results to the back. We also found lower pain thresholds at the hand expect for PPT, i.e. a site clear distinct and somatotopically far away from the painful back. This finding is of principal importance. It demonstrates increased pain sensitivity far beyond the painful lumbar back. To our knowledge, data on extrasegmental spread are not published yet. This result differs considerably from those of Blumenstiel et al. [16]. A possible explanation for this difference might lie in different sites of investigation. Blumenstiel et al. (2011) used hand dorsum (vs. hand palmar in our study). We recently found decreased pain thresholds at the lower back and the hand to punctate pinprick stimulation [13], so our previous results are in line with the data reported here. The decrease of pain thresholds at a site distinct from their region of pain (hand) might only be interpreted as a sign of centrally mediated hyperalgesia. From animal models it is known that centrally mediated hyperalgesia might involve different levels of the neuraxis, e.g. the spinal dorsal horn, thalamus, and amygdala [18]–[21]. Widespread mechanical hyperalgesia and allodynia has been found in more severe cases of migraine in human patients [18], [22] where facial mechanical allodynia spreads from the affected to the contralateral side. This was accompanied by enhanced thalamic transmission [22]. Thus a potential mechanism for this widespread pain might be plasticity at supraspinal levels, for which the thalamus is a prime candidate, since it forms the next relay of the ascending pathway. Here receptive fields can encompass whole quadrants or body sides [23]. Several findings in animals support the involvement of the thalamus; plasticity to nociceptive processing was found in the spinal or medullary dorsal horn and in the thalamus [24], [25]. Hyperalgesia was also preserved in the amygdala receiving ascending projections from the medial thalamus, as well as descending input from the anterior cingulate cortex and insula [19]. An alternative explanation might be a specific susceptibility to painful stimulation. Recently, Pfau et al. [26] reported that a part of their control subjects showed an unusual long hyperexcitability to painful stimulation after the induction of an experimental long-term potentiation (LTP)-like hyperalgesia. Such a susceptibility to high-frequency stimulation might be a predisposition for development of more widespread sensitivity. Eventually, a part of these control subjects might be affected by back pain. It is possible that the back might just be the site of primary affection. If this consideration is true then it might be that the CLPB patients are patients that suffer from back pain, but show higher pain sensitivity independently the exact site of pain, i.e. just the picture we demonstrated here. Such vulnerability might be due to hypersensitivity within the nociceptive system or due to a deficit of the endogenous antinociceptive system [27]. To differentiate between these possibilities, longitudinal studies are needed.

### Detection thresholds to innocuous stimuli

Parallel to the facilitation of mechanical painful stimuli, we identified a generalized hyposensitivity to innocuous stimuli in CLBP patients (main effect of Detection threshold). A similar hyposensitivity can be induced dynamically in experimental models and parallel to the experimental induction of central sensitization in pain patients; it resolves upon recovery [28]–[32]. Our main effect was mainly carried by the thermal detection thresholds. An increase of warmth detection threshold was also found in patients with non-neuropathic pain (including CLBP patients) [33], which was correlated to the level of on-going pain. In line with Agostinho et al. [33] we propose that persistent pain might lead to a centrally mediated impairment of non-painful thermal percept as shown previously in clinical and experimentally-induced pain [30], [34], [35]. Additionally, ongoing pain might reallocate attentional and/or working memory resources [36], [37] or processing of pain-related material [38], [39] possibly resulting in increases of sensory thresholds [40], [41].

Unexpectedly, we found that innocuous cold stimuli at the back elicited a painful sensation, i.e. paradoxical heat sensation (PHS, Fig. 1 B), in 3 out of 18 patients (Table 1). Experimentally, PHS can be induced by selective conduction blockade in A-fibers, and it is facilitated by peripheral sensitization experimentally [42]–[44]. Similar changes have been previously reported at the hand in patients with fibromyalgia, peripheral arterial occlusion, acute complex regional pain syndrome, and postherpetic neuralgia [16], [42], [45]–[47]. PHS together with a hyperalgesia to mechanical stimuli are interpreted as signs of a deficiency of pain inhibitory systems and/or altered integration of somatosensory stimuli [43], [46], [48], [49]. This result also supports the proposed mechanisms for the observed changes of pain thresholds mentioned above.

### Limitations and further directions

Our sample size with 34 subjects is relatively small. So the study should be extended to larger sample sizes and different centers. However, the sensory changes in this small sample are robust exhibiting large effect sizes (e.g., Cohen's d = 1.12 and d = 0.98 for MPS at the back and the hand, respectively; d = 0.80 and d = 1.05 for WDT at the back and the hand, respectively). Furthermore, we only investigated female subjects. Further research should clarify whether similar changes can be found in male CLBP patients.

The data do not allow to distinguish between the two major hypothesis for this higher-order effect, i.e., sensitization after an aversive event vs. susceptibility. Longitudinal studies might help to solve this question.

Our results of widespread somatosensory disturbances for noxious and innocuous stimuli might be important for the therapy of chronic low back pain. They call for a therapy that is not restricted to the painful area. Taking our results into account, they are in line with the results of clinical studies demonstrating positive effects of multidisciplinary pain management programs [50]–[52] or multisegmental approaches [53] in CLBP patients.

## Conclusion

Widespread changes of somatosensory sensitivity were found in CLBP patients. Most important, significantly enhanced pain thresholds were found not only at the back, but also at a non-painful remote site (hand). In addition, we found a significant loss of sensitivity to innocuous stimuli, especially for thermal detection. This results points to changes in the somatosensory information processing in CLBP with higher order plasticity rather than spinal cord mechanisms.

## Materials and Methods

### Participants

Eighteen chronic low back pain (CLBP) patients and sixteen pain-free healthy controls (HC) participated in this study. Healthy control subjects and CLBP patients were matched concerning age and gender (for subjects' characteristics - see Table 3). CLBP patients met the following inclusion/exclusion criteria: 1. A minimum of 6 months history of low back pain; 2. pain had been classified as ‘non-specific low back pain’ (no indicators for nerve root problems, e.g. unilateral leg pain, radiating to foot or toes, numbness and/or paraesthesia; straight leg raising test induces leg pain); 3. magnetic resonance imaging (MRI) of the spine showed only age-related changes, but no spinal disorders or disc pathology; 4. no psychiatric disorders and no disease associated to small fibre pathology (e.g.; diabetes mellitus) according to clinical anamnesis. All participants were screened for their eligibility by a female clinician (B.S.). All participants were right handed. They gave written informed consent to procedures approved by local ethics committee of the University of Jena.

**Table 3:** Characteristics of chronic low back pain patients (CLBP) and healthy controls (HC).

	CLBP (N = 18)	HC (N = 16)	P-value CLBP vs. HC
***Gender***	female	female	–
***Age*** (years):			
mean±SD	51.2±4.2	51.1±5.5	>0.05
***Employment***	all employed	all employed	–
***Duration of back pain***:			
months; median(range)	158 (6–360)	0.0	<0.001
***Back Pain Intensity before QST***			
(VAS): mean±SD	28.1±16.9	0.0±0.0	<0.001
***Average back pain intensity***			
last 4 weeks (VAS): mean±SD	34.4±13.8	0.0±0.0	<0.001
***Maximum back pain intensity***			
last 4 weeks (VAS): mean±SD	55.6±23.6	0.0±0.0	<0.001
***BDI score (depression)***:			
mean±SD	8.5±5.2	1.6±1.5	<0.001
***RDQ score (disability)***:			
mean±SD	5.0±3.4	0.1±0.5	<0.001

VAS: visual analog scale (0–100) with 0 =  ‘no pain’ and 100 =  ‘pain as bad as you can imagine’; Average back pain intensity last 4 weeks, pain intensity rating in response: ‘‘How would you rate your average back pain over the last four weeks?’’; Maximum (Max.) back pain intensity last 4 weeks, pain intensity rating in response to: ‘‘How would you rate your maximum back pain over the last four weeks? BDI: Beck Depression Inventory [57], [58]; RDQ: Roland and Morris disability Questionnaire [54]–[56]. SD: standard deviation. QST: Quantitative sensory testing.

### Medication

Medication for ten of CLBP patients was limited to non-steroidal anti-inflammatory drugs on demand (ibuprofen, diclofenac). One CLBP patient reported to take a non-opioid analgesic (katadolon) on demand, but not during the last week before the investigation. All investigated CLBP patients were without any analgesic medication for at least 48 hours before the examination.

### Disability and Depression Score

Low back pain related disability was measured using the German version of the 24-items Roland Morris disability questionnaire (RDQ) [54]–[56]. RDQ scores range from 0 (no disability) to 24 (maximum disability). Back pain intensity was measured using a VAS with 0 =  ‘no pain’ and 100 =  ‘pain as bad as you can imagine’ directly before the QST examination for the actual pain as well as for average/maximum pain over the last 4 weeks. Depression Scores were assessed using a German version [57] of the Beck Depression inventory (BDI) [58]. Table 3 shows mean characteristics of these parameters. There are some aspects to be mentioned here. Ten of the CLBP patients showed clinically relevant disability (3<RDQ score <8), one patient reported a high level of disability (RDQ>7); none of the control subject reported clinically relevant disability. Eleven of the CLBP patients showed no clinically relevant BDI scores (<10), five CLPB patients reported light depressive syndrome (score value: 10–17), two patients reported a BDI score up to 20 (moderate depressive symptoms). None of the healthy subjects reported a BDI score higher than 2.

### Quantitative sensory testing protocol

Quantitative sensory testing (QST) was assessed according to the standardized protocol of the German Research Network on Neuropathic Pain (DFNS) [14], [15]. QST was performed by a DFNS-trained investigator. The QST profile consisted of seven tests measuring 13 parameters. The parameters can be grouped as follows: detection thresholds (thermal: cold detection threshold (CDT), warm detection threshold (WDT), thermal sensory limen (TSL); mechanical: mechanical detection threshold (MDT) to von-Frey hair stimulation, vibration detection threshold (VDT)) and pain thresholds (thermal: cold pain threshold (CPT), heat pain threshold (HPT); mechanical: mechanical pain threshold (MPT) to pin prick stimuli, mechanical pain sensitivity (MPS) as an integrated parameter for pinprick stimuli with increasing intensity; pressure pain thresholds (PPT) to blunt pressure, wind-up ratio (WUR) as the summation to repetitive pinprick stimuli. Additionally, QST investigates the number of subjects showing a dynamic mechanical allodynia (DMA) or paradoxical heat sensations (PHS).

QST protocol was performed on the painful body site (paraspinal lumbar; location of measurement: vertebra Th12 to L5) and on a non-painful body site (hand palmar) in both CLBP patients and healthy control subjects. Hand palmar was used since we were also interested in the somatosensory sensibility of this region: External perturbations applied over the hand palmar were associated with prolonged reflex response latencies in CLBP. We also assessed MPS at the hand dorsum to assess our data with respect to the normative data of DFNS.

In order to exclude a potential influence of different skin temperatures of the thermal detection and thermal pain thresholds as well as all other QST parameters, skin temperature was assessed for all body sites (hand palmar, hand dorsum, back paraspinal lumbar) before and after the QST. There were no significant skin temperature changes for any of the body sites before vs. after the QST protocol or between patients and controls.

### Thermal detection thresholds and the number of paradoxical heat sensations

Thermal testing was performed using a thermal stimulator (Pathway, Model ATS, Medoc, Israel) with a thermode contact area of 9 cm^2^. Cold detection threshold (CDT), warm detection threshold (WDT), thermal sensory limen procedure (TSL, the difference limen for altering cold and warm stimuli), and the number of paradoxical heat sensations by using the, TSL were assessed using the standard protocol of DFNS (baseline temperature: 32°C, ramp rate for all thermal stimuli: 1°C/s) [14], [15].

### Mechanical detection thresholds for touch (MDT) and vibration (VDT)

Mechanical detection threshold (MDT) for light touch was assessed by using a series of standardised von Frey filaments (diameter 0.5 mm, Optihair_2_-Set Marstock Nervtest, Germany) which exerts forces between 0.25 and 512 mN (factor 2 progression). Using the ‘methods of limit’, the final threshold was assessed as the geometric mean of five series of ascending and descending stimulus intensities [59].

Vibration detection threshold (VDT) on the hand dorsum was measured using a Rydel–Seiffer graded tuning fork (64 Hz, 8/8 scale) that was placed over the processus styloideus ulnae according to the protocol of DFNS [14], [17]. For an additional examination on the back, the tuning fork was placed over three processi vertebrae at the painful site in the region of CLBP. VDT was determined as the average of three consecutive processi vertebrae around the painful site (e.g., L2-L4 for L3). VDT was determined as the disappearance threshold of vibration sensation reported by the subject.

### Thermal pain thresholds

Cold and heat thermal pain thresholds (CPT and HPT, respectively) were assessed the according to the standard protocol of DFNS using the same thermal stimulator as for detection thresholds (Pathway, Model ATS, Medoc, Israel; thermode contact area: 9 cm^2^).

### Mechanical pain threshold (MPT)

MPT was measured using standard pinprick stimulators (cylindrical tip, 250 µm tip diameter) with fixed stimulus intensities that exerted forces of 8, 16, 32, 64, 128, 256, and 512 mN (MRC Systems GmbH, Heidelberg, Germany). The Stimulators were applied in ascending order until the first percept of sharpness was detected. MPT was determined using the ‘methods of limits’. The final threshold was the geometric mean of five series of ascending and descending stimuli intensities.

### Mechanical pain sensitivity (MPS) and dynamic mechanical allodynia (DMA)

Pain induced to punctate mechanical stimuli was measured using the same standard pinprick stimulators as for MPT. To obtain MPS for pinprick-evoked pain, all seven pinprick stimuli were applied in balanced order, five times each stimuli in every test site (hand dorsum, hand palmar, paraspinal lumbar). MPS was assessed as the geometric mean of the given stimuli as in the standard protocol. To avoid effects of sensitization or fatigue, the successive stimuli were not applied at the same spot of skin, but some mm apart from previous stimulation side. Following each stimulus, participants were asked to rate the experienced pain intensity for each stimulus on a verbal rating scale (with 0 indicating ‘no pain’, and 100 indicating ‘maximal imaginable pain’). Pain to light touch (DMA, dynamic mechanical allodynia) was assessed by light stroking with a cotton wisp (3 mN), a Q-tip fixed to an elastic strip (100 mN), and a soft make-up brush (200–400 mN). The set of the three light tactile stimulators where intermingled with the pinprick stimuli in balanced order [60].

In case the stroking stimuli were perceived as painful, participants were asked to give a verbal rating for the perceived pain magnitude (0–100).

### Wind-up ratio – the temporal pain summation to repetitive pinprick stimuli (WUR)

The perceptual correlate of temporal pain summation to repetitive pinprick stimuli (WUR, wind-up ratio) was assessed by trains of ten punctate stimuli (256 mN tested over hand and back) 1 Hz repetition rates. The participants were asked to give a pain rating representing the pain at the end of the train using a numerical rating scale. The pain ratings to single pinprick stimulation were compared with those of ten repeated punctate stimuli. To determine the wind-up-ratio, the ratio of the mean pain rating of trains divided by the mean pain rating to a single stimulus was calculated.

### Pressure pain thresholds (PPT)

PPT was performed over the muscle on the painful (paraspinal lumbar) and at the non-painful site (thenar eminence palmar) using a pressure gauge device (Somedic AB, Hörby, Sweden) with a probe site of 1 cm^2^ (probe diameter of 1.1 cm) that exerts pressure up to 200 N/cm^2^/∼2000 kPa. The pressure pain threshold was determined with three series of ascending stimulus intensities, each applied as an increasing ramp of 10 kp/s.

### Data evaluation

Tests were performed depending on data distribution properties. Cold pain thresholds, heat pain thresholds and vibration detection thresholds were normally distributed as analysed by the Shapiro-Wilks test. All other parameters were normally distributed in log space, and thus they were log_10_-transformed before statistical analysis {Rolke 2006a}. Furthermore, QST data were z-transformed using the following expression:

Z-score  =  (single _individual CLBP patient_ – mean _controls_)/SD _controls_

QST z-scores of the painful and non-painful body site of each CLBP patient were compared with the group means of healthy controls using z-score. Z-scores above ‘0’ indicate a gain of function referring to the higher sensitivity of the CLBP patient to the tested stimuli compared to the healthy controls. Z-scores below ‘0’ indicate a loss of function when the CLBP patient is less sensitive to the tested stimuli compared the healthy controls. Z-values below −1.96 or above +1.96 were considered as abnormal for diagnostic purposes (95% confidence interval [17], [61]).

According to our hypothesis, we tested for significant differences for pain thresholds between the groups for both body sites using mixed-model two-way analysis of variance (ANOVA) using the z-values with respect to the group of healthy controls. We used the between-subject factor Group (CLBP patients; healthy controls) and the within-subject factors Region (paraspinal lumbar; hand palmar) and Pain threshold (CPT; HPT; MPT; MPS; PPT; WUR). We also performed a similar ANOVA for the detection thresholds using the factors Group, Region, and Detection threshold (CDT; WDT; TSL; MDT; VDT). Results were corrected for violations of sphericity using the Greenhouse-Geisser approach for ε-correction of degrees of freedom. Post-hoc analyses were performed using one-way ANOVAs and t-tests. All statistical calculations were performed using SPSS 19 Software. Data are presented as mean z-scores of QST parameters±standard error of the mean (SEM).

MPS was assessed at the hand dorsum in order to compare these data with the reference data from the German Research Network on Neuropathic Pain (DFNS) [17]. As proposed recently [17], a t-test (two-sided for independent samples) was performed using the internet-based statistical freeware Simple Interactive Statistical Analysis (SISA; URL: http://www.quantitativeskills.com/sisa/) separately for healthy controls and CLBP patients to compare data with the DFNS reference data.

## Supporting Information

Table S1 **Individual values from the Quantitative sensory testing at the painful site (back, paraspinal lumbar) in female chronic low back pain patients (CLBP) and female healthy controls (HC).**
(PDF)

Table S2 **Individual values from the Quantitative sensory testing for the site distinct from the painful region (hand palmar) in female chronic low back pain patients (CLBP) and female healthy controls (HC).**
(PDF)

Table S3 **Individual values from the Quantitative sensory testing for the site distinct from the painful region (hand dorsum) in female chronic low back pain patients (CLBP) and female healthy controls (HC).**
(PDF)
