# Supplementary material for: Somatosensory Abnormalities for Painful and Innocuous Stimuli at the Back and at a Site Distinct from the Region of Pain in Chronic Back Pain Patients
Source: PLoS One. 2013 Mar 15;8(3):e58885. doi: 10.1371/journal.pone.0058885 (PMC3598908; doi:10.1371/journal.pone.0058885)
Supplement: Table S3 — Individual values from the Quantitative sensory testing for the site distinct from the painful region (hand dorsum) in female chronic low back pain patients (CLBP) and female healthy controls (HC). (PDF) [file pone.0058885.s003.pdf]

Table S3. Individual values from the Quantitative sensory testing for the site distinct from the painful region (hand dorsum) in female chronic low back pa patients (CLBP) and female healthy controls (HC).

Subjects	Nr.	MPS	MPS_ log_{10}	VDT	DMA	DMA_ log_{10}
CLBP	1	2.75	0.455	6.53	0.00	-1.00
CLBP	2	0.38	-0.319	6.70	0.00	-1.00
CLBP	3	0.14	-0.620	7.23	0.00	-1.00
CLBP	4	7.79	0.897	6.90	0.00	-1.00
CLBP	5	3.15	0.512	6.20	0.00	-1.00
CLBP	6	2.40	0.398	6.53	0.00	-1.00
CLBP	7	2.12	0.346	6.43	0.00	-1.00
CLBP	8	2.05	0.332	7.37	0.00	-1.00
CLBP	9	5.41	0.741	6.20	0.00	-1.00
CLBP	10	5.61	0.757	7.57	0.00	-1.00
CLBP	11	9.41	0.978	6.77	0.00	-1.00
CLBP	12	3.66	0.575	6.17	0.02	-0.92
CLBP	13	0.69	-0.102	7.27	0.00	-1.00
CLBP	14	1.55	0.217	7.77	0.00	-1.00
CLBP	15	0.60	-0.155	6.90	0.00	-1.00
CLBP	16	0.61	-0.149	6.87	0.00	-1.00
CLBP	17	9.99	1.004	7.60	0.22	-0.49
CLBP	18	2.22	0.365	7.43	0.00	-1.00
HC	1	1.55	0.217	7.83	0.00	-1.00
HC	2	0.16	-0.585	7.10	0.00	-1.00
HC	3	0.30	-0.398	6.13	0.00	-1.00
HC	4	1.36	0.164	7.77	0.00	-1.00
HC	5	1.60	0.230	6.00	0.00	-1.00
HC	6	0.67	-0.114	6.93	0.00	-1.00
HC	7	4.16	0.629	7.70	0.00	-1.00
HC	8	5.49	0.747	6.10	0.00	-1.00
HC	9	0.67	-0.114	6.40	0.00	-1.00
HC	10	0.77	-0.060	7.03	0.00	-1.00
HC	11	0.94	0.017	7.30	0.00	-1.00
HC	12	0.37	-0.328	7.90	0.00	-1.00
HC	13	0.88	-0.009	6.93	0.00	-1.00
HC	14	0.13	-0.638	7.53	0.00	-1.00
HC	15	0.12	-0.658	7.07	0.00	-1.00
HC	16	0.16	-0.585	6.77	0.00	-1.00

MPS: Mechanical pain sensitivity; VDT: Vibration detection threshold; DMA: Dynamic mechanical allodynia.
log_{10}: log_{10} transformation of original data.
