# Supplementary material for: Somatosensory Abnormalities for Painful and Innocuous Stimuli at the Back and at a Site Distinct from the Region of Pain in Chronic Back Pain Patients
Source: PLoS One. 2013 Mar 15;8(3):e58885. doi: 10.1371/journal.pone.0058885 (PMC3598908; doi:10.1371/journal.pone.0058885)
Supplement: Table S1 — Individual values from the Quantitative sensory testing at the painful site (back, paraspinal lumbar) in female chronic low back pain patients (CLBP) and female healthy controls (HC). (PDF) [file pone.0058885.s001.pdf]

Table S1. Individual values from the Quantitative sensory testing at the painful site (back, paraspinal lumbar) in female chronic low back pain patients (CLBP) and female healthy controls (HC).

Subjects	Nr.	CDT	CDT_log$_{10}$	WDT	WST_ log$_{10}$	TSL	TSL_ log$_{10}$	CPT	HPT	PPT	PPT_ log$_{10}$	MPT	MPT_ log$_{10}$	MPS	MPS_ log$_{10}$	WUR	WUR_ log$_{10}$	MDT	MDT_ log$_{10}$	VDT	DMA	DMA_ log$_{10}$	PHS
CLBP	1	-0.90	-0.046	1.77	0.248	4.08	0.611	28.47	35.07	22	1.342	15.00	1.176	4.52	0.665	7.85	0.895	9.85	0.993	4.70	0.00	-1.00	0
CLBP	2	-1.10	0.041	3.73	0.572	5.93	0.773	20.40	45.83	137	2.137	7.00	0.845	0.37	-0.328	1.90	0.279	6.96	0.843	4.57	0.00	-1.00	0
CLBP	3	-3.70	0.568	3.57	0.553	8.53	0.931	1.00	46.43	168	2.225	11.00	1.041	0.17	-0.569	1.33	0.124	4.59	0.662	5.67	0.00	-1.00	0
CLBP	4	-1.53	0.185	2.53	0.403	5.24	0.719	21.6	44.93	243	2.386	7.00	0.845	12.54	1.102	2.39	0.378	9.85	0.993	5.50	0.00	-1.00	0
CLBP	5	-6.90	0.839	2.37	0.375	15.79	1.198	13.30	39.43	110	2.041	7.00	0.845	3.72	0.582	1.36	0.134	11.31	1.053	4.40	0.00	-1.00	3
CLBP	6	-8.33	0.921	4.83	0.684	9.97	0.999	1.00	41.80	121	2.083	6.00	0.778	1.38	0.170	1.64	0.215	1.15	0.061	3.90	0.00	-1.00	0
CLBP	7	-1.00	0.000	1.57	0.196	2.97	0.473	1.10	35.80	110	2.041	9.85	0.993	6.08	0.791	2.57	0.410	2.00	0.301	3.50	0.00	-1.00	0
CLBP	8	-1.30	0.114	1.77	0.248	5.66	0.753	23.20	40.83	121	2.083	13.00	1.114	2.78	0.459	1.86	0.270	0.31	-0.509	6.23	0.00	-1.00	1
CLBP	9	-1.07	0.029	1.43	0.155	3.53	0.548	21.97	46.13	80	1.903	7.46	0.873	13.84	1.144	3.14	0.497	1.41	0.149	5.37	0.27	-0.43	3
CLBP	10	-1.80	0.255	1.93	0.286	3.79	0.579	21.37	41.33	239	2.378	6.50	0.813	6.00	0.785	5.38	0.731	0.54	-0.268	5.53	0.00	-1.00	0
CLBP	11	-1.10	0.041	1.93	0.286	3.21	0.507	17.03	38.37	148	2.170	6.50	0.813	14.94	1.177	1.79	0.253	4.00	0.602	5.13	0.00	-1.00	0
CLBP	12	-2.17	0.336	1.93	0.286	5.74	0.759	2.630	42.80	133	2.124	5.66	0.753	4.99	0.707	3.09	0.49	6.50	0.813	3.53	0.03	-0.89	0
CLBP	13	-1.47	0.167	2.20	0.342	4.83	0.684	25.13	39.13	169	2.228	29.86	1.475	1.95	0.312	1.63	0.212	1.74	0.241	5.23	0.00	-1.00	0
CLBP	14	-6.83	0.834	4.47	0.65	14.38	1.158	1.00	45.20	400	2.602	5.66	0.753	6.27	0.804	2.97	0.473	1.23	0.09	6.73	0.00	-1.00	0
CLBP	15	-4.10	0.613	1.97	0.294	7.33	0.865	17.90	43.97	331	2.520	6.50	0.813	0.56	-0.18	2.91	0.464	2.46	0.391	4.33	0.00	-1.00	0
CLBP	16	-0.60	-0.222	2.53	0.403	8.02	0.904	5.50	44.20	278	2.444	12.13	1.084	0.52	-0.208	3.10	0.491	3.03	0.481	5.50	0.00	-1.00	0
CLBP	17	-1.40	0.146	2.47	0.393	4.27	0.63	1.00	41.93	196	2.292	11.31	1.053	12.54	1.102	1.68	0.225	1.23	0.09	5.03	0.09	-0.72	0
CLBP	18	-2.27	0.356	1.77	0.248	4.98	0.697	23.33	39.77	190	2.279	6.96	0.843	3.47	0.553	3.56	0.551	0.54	-0.268	5.57	0.00	-1.00	0
HC	1	-0.57	-0.244	1.43	0.155	3.17	0.501	9.30	44.00	121	2.083	19.70	1.294	1.29	0.143	9.00	0.954	0.81	-0.092	6.77	0.00	-1.00	0
HC	2	-4.13	0.616	4.27	0.63	10.82	1.034	11.23	44.43	140	2.146	14.93	1.174	0.17	-0.569	1.57	0.196	6.5	0.813	3.20	0.00	-1.00	0
HC	3	-1.30	0.114	2.63	0.42	5.18	0.714	1.00	45.03	289	2.461	6.50	0.813	0.12	-0.658	14.00	1.146	4.92	0.692	4.77	0.00	-1.00	0
HC	4	-0.90	-0.046	1.63	0.212	5.13	0.71	12.00	45.20	200	2.301	12.13	1.084	1.17	0.104	2.67	0.427	1.23	0.09	5.57	0.00	-1.00	0
HC	5	-1.17	0.068	1.23	0.090	3.73	0.572	24.67	39.20	200	2.301	11.31	1.053	1.23	0.124	2.75	0.439	6.06	0.782	4.97	0.00	-1.00	0
HC	6	-2.47	0.393	1.17	0.068	4.39	0.642	8.93	44.27	205	2.312	9.85	0.993	0.96	0.025	5.00	0.699	0.54	-0.268	5.33	0.00	-1.00	0
HC	7	-0.87	-0.060	1.13	0.053	2.22	0.346	1.00	44.13	83	1.919	25.99	1.415	6.35	0.810	7.96	0.901	1.32	0.121	5.97	0.00	-1.00	0
HC	8	-1.00	0.000	1.53	0.185	3.44	0.537	16.57	45.07	195	2.290	6.96	0.843	5.96	0.782	1.53	0.185	1.87	0.272	4.83	0.00	-1.00	0
HC	9	-1.47	0.167	1.33	0.124	3.65	0.562	13.90	39.30	116	2.064	9.85	0.993	2.19	0.360	4.69	0.671	1.15	0.061	5.33	0.00	-1.00	0
HC	10	-1.17	0.068	2.27	0.356	4.74	0.676	14.47	45.87	161	2.207	16.00	1.204	1.50	0.204	3.60	0.556	2.46	0.391	5.70	0.00	-1.00	0
HC	11	-1.27	0.104	2.03	0.307	4.27	0.63	17.80	40.97	199	2.299	24.25	1.385	0.73	-0.081	1.40	0.146	3.03	0.481	4.53	0.00	-1.00	0
HC	12	-2.80	0.447	2.50	0.398	6.71	0.827	7.63	48.00	218	2.338	5.66	0.753	0.25	-0.456	6.00	0.778	2.14	0.330	5.10	0.00	-1.00	0
HC	13	-1.40	0.146	1.97	0.294	3.73	0.572	6.17	42.33	218	2.338	21.11	1.324	1.12	0.086	1.98	0.297	2.83	0.452	3.90	0.00	-1.00	0
HC	14	-1.03	0.013	1.20	0.079	3.39	0.53	1.00	44.20	420	2.623	12.13	1.084	0.12	-0.658	2.00	0.301	1.23	0.090	6.47	0.00	-1.00	0
HC	15	-1.23	0.090	2.10	0.322	4.53	0.656	9.57	47.10	430	2.633	9.85	0.993	0.05	-0.824	1.00	0.000	2.00	0.301	5.57	0.00	-1.00	0
HC	16	-1.63	0.212	1.57	0.196	2.65	0.423	1.00	46.77	241	2.382	6.96	0.843	0.09	-0.721	4.07	0.610	3.03	0.481	5.71	0.00	-1.00	0

Thermal detection thresholds (CDT. WDT) are expressed as the difference from baseline temperature (BL = 32°C).

CDT: Cold detection threshold; WDT: Warm detection threshold; TSL: Thermal sensory limen; CPT: Cold pain threshold; HPT: Heat pain threshold; PPT: Pressure pain threshold; MPT: Mechanical pain threshold; MPS: Mechanical pain sensitivity; WUR: Wind-up ratio; MDT: Mechanical detection threshold;

VDT: Vibration detection threshold; DMA: Dynamic mechanical allodynia; PHS: Paradoxical heat sensations. log$_{10}$: log$_{10}$ transformation of original data.
