# Supplementary material for: Somatosensory Abnormalities for Painful and Innocuous Stimuli at the Back and at a Site Distinct from the Region of Pain in Chronic Back Pain Patients
Source: PLoS One. 2013 Mar 15;8(3):e58885. doi: 10.1371/journal.pone.0058885 (PMC3598908; doi:10.1371/journal.pone.0058885)
Supplement: Table S2 — Individual values from the Quantitative sensory testing for the site distinct from the painful region (hand palmar) in female chronic low back pain patients (CLBP) and female healthy controls (HC). (PDF) [file pone.0058885.s002.pdf]

Table S2. Individual values from the Quantitative sensory testing for the site distinct from the painful region (hand palmar) in female chronic low back pain patients (CLBP) and female healthy controls (HC).

Subjects	Nr.	CDT	CDT_log$_{10}$	WDT	WST_ log$_{10}$	TSL	TSL_ log$_{10}$	CPT	HPT	PPT	PPT_ log$_{10}$	MPT	MPT_ log$_{10}$	MPS	MPS_ log$_{10}$	WUR	WUR_ log$_{10}$	MDT	MDT_ log$_{10}$	DMA	DMA_ log$_{10}$	PHS
CLBP	1	-1.00	0.000	2.03	0.3075	2.70	0.431	19.03	35.53	393	2.594	34.00	1.531	5.76	0.768	5.82	0.765	2.3	0.362	0.00	-1.00	0
CLBP	2	-1.27	0.104	2.10	0.3222	3.87	0.588	8.13	51.25	347	2.540	104.00	2.017	0.16	-0.585	1.29	0.111	0.81	-0.092	0.00	-1.00	0
CLBP	3	-3.23	0.509	5.00	0.6990	7.96	0.901	1.00	50.70	398	2.600	26.00	1.415	0.29	-0.409	1.00	0.000	0.71	-0.149	0.00	-1.00	0
CLBP	4	-2.30	0.362	3.93	0.5944	5.14	0.711	6.77	41.40	583	2.766	23.00	1.362	6.62	0.827	1.28	0.107	0.31	-0.509	0.00	-1.00	0
CLBP	5	-2.93	0.467	3.80	0.5798	6.05	0.782	6.60	43.73	349	2.543	32.00	1.505	3.08	0.502	1.00	0.000	0.38	-0.420	0.02	-0.92	0
CLBP	6	-2.50	0.398	2.00	0.3010	4.13	0.616	11.23	42.93	318	2.502	28.00	1.447	1.21	0.117	1.13	0.053	0.35	-0.456	0.00	-1.00	0
CLBP	7	-1.87	0.272	1.90	0.2788	3.92	0.593	1.83	44.17	92	1.964	90.51	1.957	1.39	0.173	2.10	0.322	0.18	-0.745	0.00	-1.00	0
CLBP	8	-1.37	0.137	1.40	0.1461	2.32	0.365	1.00	44.27	121	2.083	39.40	1.595	1.8	0.279	2.06	0.314	0.18	-0.745	0.00	-1.00	0
CLBP	9	-1.20	0.079	1.40	0.1461	2.27	0.356	20.30	35.87	145	2.161	14.93	1.174	6.96	0.849	2.14	0.330	0.71	-0.149	0.00	-1.00	0
CLBP	10	-1.57	0.196	2.43	0.3856	3.58	0.554	7.03	48.60	237	2.375	36.76	1.565	3.79	0.590	1.91	0.281	0.47	-0.328	0.00	-1.00	0
CLBP	11	-1.93	0.286	1.77	0.2480	2.42	0.384	8.50	38.20	180	2.255	34.30	1.535	3.83	0.594	1.97	0.294	0.31	-0.509	0.00	-1.00	0
CLBP	12	-1.50	0.176	1.17	0.0682	2.78	0.444	8.23	39.43	155	2.190	36.76	1.565	1.84	0.288	4.00	0.602	0.47	-0.328	0.00	-1.00	0
CLBP	13	-1.43	0.155	1.07	0.0294	2.44	0.387	15.83	40.43	196	2.292	68.59	1.836	0.51	-0.215	1.87	0.272	0.18	-0.745	0.00	-1.00	0
CLBP	14	-3.67	0.565	2.67	0.4265	4.80	0.681	1.00	49.30	346	2.539	42.22	1.626	1.99	0.320	1.67	0.223	0.19	-0.721	0.00	-1.00	0
CLBP	15	-1.87	0.272	1.87	0.2718	3.47	0.540	1.00	52.10	310	2.491	9.85	0.993	0.22	-0.495	6.17	0.790	0.27	-0.569	0.00	-1.00	0
CLBP	16	-0.83	-0.081	0.70	-0.1549	1.90	0.279	1.10	46.50	149	2.173	25.99	1.415	1.64	0.241	2.69	0.430	0.22	-0.658	0.00	-1.00	0
CLBP	17	-2.07	0.316	2.00	0.3010	3.71	0.569	1.00	48.93	141	2.149	21.11	1.324	6.85	0.842	2.42	0.384	0.18	-0.745	0.22	-0.49	0
CLBP	18	-1.47	0.167	0.90	-0.0458	2.93	0.467	0.93	47.60	350	2.544	42.22	1.626	1.42	0.182	4.75	0.677	0.35	-0.456	0.00	-1.00	0
HC	1	-0.87	-0.060	0.93	-0.032	4.06	0.609	13.80	47.27	156	2.193	19.70	1.294	1.18	0.107	2.14	0.330	0.18	-0.745	0.00	-1.00	0
HC	2	-1.27	0.104	1.60	0.204	3.19	0.504	9.00	45.17	233	2.367	13.93	1.144	0.43	-0.276	1.75	0.243	0.38	-0.420	0.00	-1.00	0
HC	3	-1.10	0.041	1.20	0.079	2.14	0.330	5.17	41.50	178	2.250	84.45	1.927	0.39	-0.310	2.83	0.452	0.35	-0.456	0.00	-1.00	0
HC	4	-0.97	-0.013	1.47	0.167	2.41	0.382	3.93	36.77	206	2.314	12.13	1.084	0.91	0.004	1.74	0.241	0.35	-0.456	0.02	-0.92	0
HC	5	-2.47	0.393	1.73	0.238	3.42	0.534	8.47	42.67	159	2.201	51.98	1.716	0.99	0.037	1.93	0.286	0.66	-0.180	0.00	-1.00	0
HC	6	-1.27	0.104	0.63	-0.201	1.37	0.137	1.00	43.53	293	2.467	111.43	2.047	0.66	-0.119	2.86	0.456	0.35	-0.456	0.00	-1.00	0
HC	7	-0.90	-0.046	1.13	0.053	2.74	0.438	1.00	51.87	112	2.049	256.00	2.408	2.92	0.480	2.59	0.413	0.18	-0.745	0.00	-1.00	0
HC	8	-1.50	0.176	0.97	-0.013	2.48	0.394	1.17	44.10	179	2.253	17.15	1.234	2.82	0.465	1.49	0.173	0.31	-0.509	0.00	-1.00	0
HC	9	-1.63	0.212	0.80	-0.097	3.02	0.480	2.40	44.63	190	2.279	45.25	1.656	0.66	-0.119	2.87	0.458	0.2	-0.699	0.00	-1.00	0
HC	10	-1.00	0.000	1.10	0.041	1.97	0.294	3.30	46.47	139	2.143	103.97	2.017	0.35	-0.347	4.40	0.643	0.18	-0.745	0.00	-1.00	0
HC	11	-1.40	0.146	1.93	0.286	3.79	0.579	1.00	42.17	274	2.438	181.02	2.258	0.52	-0.208	2.00	0.301	0.38	-0.420	0.00	-1.00	0
HC	12	-1.13	0.053	1.17	0.068	2.74	0.438	8.73	46.17	278	2.444	29.86	1.475	0.17	-0.569	3.27	0.515	0.2	-0.699	0.00	-1.00	0
HC	13	-1.00	0.000	1.07	0.029	2.28	0.358	2.00	45.80	212	2.326	90.51	1.957	0.31	-0.387	0.98	-0.009	0.35	-0.456	0.00	-1.00	0
HC	14	-1.30	0.114	1.50	0.176	3.31	0.520	1.00	50.40	453	2.656	34.30	1.535	0.22	-0.495	2.44	0.387	0.81	-0.092	0.00	-1.00	0
HC	15	-1.50	0.176	1.50	0.176	7.15	0.854	1.00	48.27	246	2.391	64.00	1.806	0.23	-0.481	2.67	0.427	0.19	-0.721	0.00	-1.00	0
HC	16	-1.60	0.204	1.20	0.079	2.90	0.462	1.00	50.23	228	2.358	97.01	1.987	0.13	-0.638	24.00	1.380	0.33	-0.481	0.00	-1.00	0

Thermal detection thresholds (CDT. WDT) are expressed as the difference from baseline temperature (BL = 32°C).

CDT: Cold detection threshold; WDT: Warm detection threshold; TSL: Thermal sensory limen; CPT: Cold pain threshold; HPT: Heat pain threshold; PPT: Pressure pain threshold; MPT: Mechanical pain threshold; MPS: Mechanical pain sensitivity; WUR: Wind-up ratio; MDT: Mechanical detection threshold;

VDT: Vibration detection threshold; DMA: Dynamic mechanical allodynia; PHS: Paradoxical heat sensations. log$_{10}$: log$_{10}$ transformation of original data.
